# Small RNAs as emerging regulators of commensal adaptation in the mammalian gut

**DOI:** 10.1128/iai.00292-26

**Published:** 2026-10-15

**Authors:** Ryan T. Fansler, Wenhan Zhu

**Affiliations:** 1 Department of Microbiology and Immunology, University of Michigan Medical School 1259, Ann Arbor, Michigan, USA; 2 Department of Pathology, Microbiology, and Immunology, Vanderbilt University Medical Center 12328, Nashville, Tennessee, USA; 3 Vanderbilt Institute for Infection, Immunology, and Inflammation, Vanderbilt University Medical Center 12328, Nashville, Tennessee, USA; University of California Merced, Merced, California, USA

**Keywords:** microbiota, commensal, sRNA, small RNA, resilience, gut microbiome, stress adaptation, bacterial

## Abstract

The commensal bacteria of the intestinal microbiota inhabit one of the most densely populated and dynamic microbial ecosystems known, where fluctuations in nutrient availability, host-derived stresses, and microbial competition require highly coordinated regulatory responses to maintain colonization and fitness. As a stable, homeostatic microbiota performs essential functions for the host, understanding how commensals adapt to the fluctuating intestinal environment is important for maintaining intestinal homeostasis and informing the development of new microbiota-targeted therapeutics. Throughout diverse bacterial species, rapid adaptation to environmental perturbations is mediated in part by small regulatory RNAs (sRNAs). While most sRNA research has focused on a limited number of model organisms, particularly bacterial pathogens, recent studies have uncovered important roles for these noncoding RNAs in promoting the fitness and resilience of gut commensals. Advances in differential RNA sequencing, comparative genomics, and functional genetics have revealed diverse repertoires of sRNAs in prominent gut symbionts, with regulatory roles in nutrient acquisition, stress responses, and host colonization. This minireview highlights recent advances and outlines future directions to further elucidate sRNA-mediated regulation as a pillar of commensal resilience in the mammalian gut.

## INTRODUCTION

The human gastrointestinal tract is inhabited by a diverse consortium of bacteria, viruses, fungi, archaea, and protozoa (1). Commensal bacteria, a key subset of the gut microbiota, perform essential functions for human health, including assisting in nutrient metabolism (2), immune system development (3–5), and resistance to invading enteric pathogens (reviewed in 6, 7). To stably persist in the intestinal environment, these commensals must remain resilient in the face of near-constant perturbations, including shifts in nutrient availability (8–10), pharmaceutical use (11–14), dietary changes (15, 16), and inflammatory insults (17, 18). Beyond these environmental challenges, commensal bacteria must also navigate intense nutrient competition with neighboring microbes, as the gut microbiota represents one of the densest microbial ecosystems known, reaching abundances of roughly 10^11^–10^12^ bacteria per gram of luminal intestinal contents (19). How the commensal microbiota responds to these pressures directly impacts host health, as it determines both their functional stability (reviewed in 20) and their potential for opportunistic pathogenesis (reviewed in 21). For example, when deprived of dietary fiber, the prominent commensal *Bacteroides* can shift to utilizing host-derived glycoproteins, leading to erosion of the intestinal mucus layer and weakening of barrier defenses against infection (22). Mucus utilization by *Bacteroides* spp. has been associated with increased intestinal inflammation and exacerbation of immune-mediated disorders, including graft-versus-host disease (23). Thus, a deeper understanding of commensal adaptation to the intestinal environment is essential for elucidating the mechanisms underlying gut health and disease.

Surviving in the highly dynamic and competitive intestinal ecosystem requires tight regulatory mechanisms that allow bacteria to rapidly remodel their transcriptomes in response to changes in nutrient availability and environmental stressors (24–27). This regulation is mediated by an impressive array of transcription factors, phase-variable promoters, DNA inversions, extracytoplasmic sigma/anti-sigma factor pairs, two-component systems (TCS) and hybrid two-component systems (HTCS), riboswitches, and small noncoding RNAs (sRNAs) (reviewed in 28). Together, these regulatory systems enable gut commensals to sense and respond to environmental cues while maintaining fitness within the intestinal ecosystem (29–31). Among these mechanisms, sRNAs have emerged as key post-transcriptional regulators of stress responses, metabolic adaptation, and virulence systems across diverse bacterial species (32–36). Unlike protein-based regulators, sRNAs can rapidly and efficiently reprogram gene expression at the post-transcriptional level, making them particularly well-suited to respond to the rapidly changing conditions encountered in the intestinal environment (37–40).

Most bacterial sRNA research in bacteria has focused on a limited number of model organisms, especially pathogens (32–34, 41–43). Until quite recently, the role of sRNA regulators in members of the commensal microbiota had remained largely unexplored. However, in recent years, new studies have uncovered a large array of sRNAs encoded by gut commensals (44, 45). This minireview will highlight these discoveries of sRNA regulators in intestinal commensal bacteria. We will discuss current approaches for identifying sRNAs in gut commensals, summarize their known regulatory functions, and examine how these molecules contribute to bacterial fitness within the mammalian intestine. Much pioneering work has been performed on the model gut symbiont *Bacteroides thetaiotaomicron (44, 46, 47*), revealing the importance of sRNA regulation in the physiology of a prominent commensal and establishing experimental and computational approaches applicable to additional members of the microbiota. As investigations expand beyond traditional model organisms (45), it is becoming clear that sRNA-mediated regulation is a pillar of commensal adaptation to the intestinal environment. Given the central role of commensal resilience in shaping both microbiota composition and host physiology (48), defining the regulatory functions of these sRNAs promises to elucidate the mechanisms that promote intestinal health and contribute to disease.

## FUNDAMENTALS OF SMALL RNA-MEDIATED REGULATION

sRNA regulators range from 50 to 500 nucleotides (49). These sRNAs can be located between encoded genes (intergenic), antisense to coding regions, or derived from the 5′ or 3′ untranslated regions (UTR) of transcripts. sRNAs typically act as post-transcriptional regulators by binding to mRNA via sequence complementarity, often aided by RNA-binding protein chaperones, to modulate transcript stability and/or translation (reviewed in 50, 51). These regulators can be designated as cis-encoded sRNAs, which bind with full complementarity to their antisense regions, or trans-encoded sRNAs that use imperfect sequence complementarity to bind targets beyond their locus of origin (52). Through these interactions, sRNAs can rapidly reshape gene expression programs involved in metabolism (53), stress adaptation (reviewed in 54), virulence (reviewed in 55), and environmental sensing (reviewed in 56), allowing bacteria to respond efficiently to changing conditions. This has been shown most extensively in model organisms and pathogens, especially *Escherichia coli* and *Salmonella* (49). These studies have revealed that sRNA-mediated regulation is a central component of bacterial regulatory networks and plays a critical role in adaptation to environmental perturbations.

**Fig 1:**
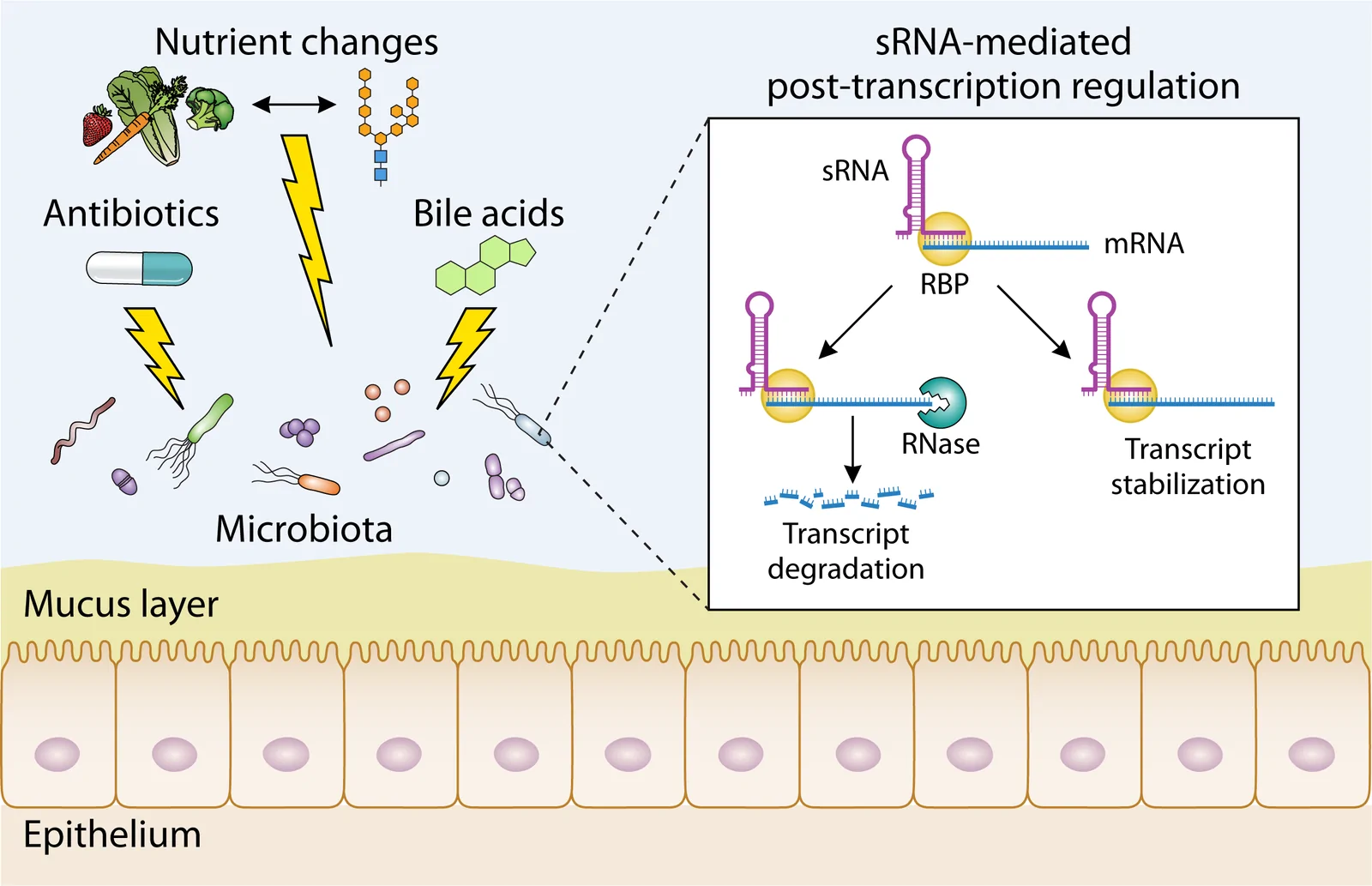
The intestinal microbiota inhabits a highly dynamic niche that is continually shaped by perturbations, including antibiotic exposure, changes in nutrient availability, bile acids, inflammation, and host-derived factors. To stably colonize this environment, commensal bacteria must rapidly and efficiently remodel their transcriptomes in response to changing conditions. Small regulatory RNAs (sRNAs) provide a rapid and energetically efficient mechanism for post-transcriptional gene regulation. Typically, sRNAs base-pair with target mRNAs through imperfect complementarity, often with the assistance of RNA-binding proteins (RBPs). Depending on the regulatory mechanism, sRNA binding can promote transcript degradation or inhibit translation, resulting in gene repression, or alternatively stabilize the transcript and/or increase ribosome accessibility, leading to enhanced gene expression.

Perhaps the most extensively characterized bacterial sRNA is RyhB, which exemplifies the conserved mechanisms of sRNA-mediated regulation and its importance to bacterial adaptation. First identified in *E. coli*, RyhB is a 90-nucleotide trans-encoded sRNA that functions as a central regulator of the bacterial iron starvation response (57). Under iron-replete conditions, RyhB transcription is repressed by iron-bound ferric uptake regulator (Fur). As intracellular iron becomes limiting, Fur-mediated repression is relieved, resulting in rapid RyhB expression. Aided by the chaperone Hfq, RyhB base-pairs with its target mRNAs to modulate their stability and translation (57). To mediate an iron-sparing program under iron-restricted conditions, RyhB targets transcripts of nonessential iron-containing proteins, thereby downregulating their expression and prioritizing iron for essential functions. For example, in concert with Hfq, RyhB binds to the *sodB* transcript, which encodes an iron-containing superoxide dismutase. RyhB binding recruits RNase E and RNase III, promoting the degradation of *sodB* mRNA and reducing its synthesis during iron limitation (58, 59). While RyhB most commonly functions as a negative regulator by promoting transcript degradation or inhibiting translation, it can also activate gene expression by stabilizing target transcripts or altering mRNA secondary structure to increase translation. RyhB directly activates expression of the *shiA* gene, which encodes a permease for shikimate, by engaging the 5′ UTR of the *shiA* mRNA and preventing formation of a secondary structure that blocks the transcript’s ribosome binding site (60). This activity promotes the import of shikimate, which is utilized in the synthesis of siderophores for iron capture during iron starvation (32). The ability of RyhB to both repress and activate gene expression enables coordinated adaptation to iron limitation and exemplifies the remarkable versatility of bacterial sRNA regulators (Fig. 1). Homologs of RyhB have been identified in numerous members of the *Enterobacterales* and related gram-negative bacteria, including *Salmonella (61*)*, Klebsiella* (62), *Yersinia* (63)*,* and *Vibrio* (64), highlighting the evolutionary conservation of this regulatory strategy for coordinating bacterial adaptation to iron availability.

The mechanistic principles established through study of RyhB and other model sRNAs have provided a foundation for investigating sRNA regulation in diverse bacterial species. sRNAs have since been found to play key roles in adaptation to oxidative stress (65–67), nitrosative stress (68), acid resistance (69), and nutrient starvation (70). Until recently, however, sRNA biology in the intestinal microbiota remained largely unexplored. Advances in differential RNA sequencing (dRNA-seq), transcriptome mapping, and genetic tools have now enabled the systematic identification and functional characterization of sRNAs in prominent gut symbionts, with *B. thetaiotaomicron* emerging as the leading model for understanding how RNA-mediated regulation contributes to nutrient acquisition, stress adaptation, and persistence within the mammalian intestine (44).

## DEFINING THE *BACTEROIDES* RNA REGULATORY LANDSCAPE

Among the most abundant genera of the gut microbiota, *Bacteroides* comprises canonical commensals that contribute to the maintenance of gut homeostasis (71) and colonization resistance (72). Its abundance, genetic tractability, and relevance to human health have made this genus a key model for studying gut commensals (73). Until recently, relatively little was known about sRNA-mediated regulation in *Bacteroides*. Only a handful of regulatory RNAs had been described, including the trans-acting sRNA RteR (74), which regulates conjugative transposon transfer, and the cis-antisense RNA DonS (75), which represses expression of a polysaccharide utilization locus (PUL) involved in host glycan metabolism. While these examples suggested that sRNA regulators contribute to *Bacteroides’* environmental adaptation, they provided little insight into the breadth or physiological significance of sRNA-mediated regulation in this prominent commensal genus. Addressing this knowledge gap, Ryan et al. used dRNA-seq, which can distinguish primary and processed 5′ ends of transcripts (76, 77), to generate the first single-nucleotide-resolution transcriptome map of *B. thetaiotaomicron (44*). In addition to defining transcriptional start sites (TSSs), UTRs, and promoter motifs in *B. thetaiotaomicron*, this study identified hundreds of candidate noncoding sRNAs in the bacterium. The putative sRNAs comprise a diverse collection of intergenic, antisense, and UTR-derived sRNAs, several of which were validated by Northern blot analysis. The resulting transcriptomic annotations were incorporated into the publicly available Theta-Base web browser, providing the first comprehensive resource for exploring gene and sRNA expression in *Bacteroides*.

Although transformative, this initial atlas was generated exclusively from bacteria grown in nutrient-rich media (44). To better reflect the dynamic nature of the gut ecosystem, Ryan et al. later expanded their transcriptome database to include *B. thetaiotaomicron* expression upon exposure to physiologically relevant environmental cues, including oxidative stress, acidic pH, bile acids, and antibiotic exposure (46). To model metabolic fluctuations in the intestinal environment, they also sampled *B. thetaiotaomicron* cultured in minimal medium supplemented with defined simple sugars or host mucus glycans. These data substantially refined transcript annotations, identified additional TSSs and noncoding RNAs, and, importantly, enabled the expression of individual sRNAs to be linked with specific environmental conditions. By integrating these transcriptomic data sets with genome-wide transposon mutant fitness data, the authors developed an atlas tying individual sRNAs to specific cellular processes (46). Together, these data provide a strong starting point for follow-up studies, serving as an atlas to prioritize conditionally important sRNAs for further functional characterization.

The dramatic expansion of the *Bacteroides* sRNA repertoire immediately raised an important mechanistic question. In nearly all well-characterized gram-negative bacteria, trans-acting sRNAs depend on global RNA chaperones, particularly Hfq (78) and ProQ (79), to facilitate RNA-RNA interactions, stabilize regulatory RNAs, and promote target recognition (80). However, comparative genomic analyses consistently failed to identify homologs of any of these canonical RNA chaperones in *Bacteroides*. By mining the *B. thetaiotaomicron* genome for proteins containing conserved RNA-binding domains, Adams et al. identified a conserved family of small proteins with a single RNA Recognition Motif (RRM-1), designated RbpA, RbpB, and RbpC (80). These proteins are widely distributed throughout the *Bacteroides* genus and are among the most highly expressed genes in *Bacteroides* species, both *in vitro* and during intestinal colonization. Biochemical analyses demonstrated that purified RbpB binds single-stranded RNA *in vitro*, with affinities comparable to established bacterial RNA-binding proteins, while transcriptomic analyses of Δ*rbp* deletion mutants revealed widespread changes in gene expression, particularly among PULs and capsular polysaccharide biosynthesis loci. While the mechanisms of action of these RBPs remained uncharacterized, this study identified an important new family of global regulatory proteins in the *Bacteroidetes* (81). Together, these findings laid the foundation for functional studies that have begun to reveal how individual sRNAs (Table 1) and their partner RNA-binding proteins promote *B. thetaiotaomicron* adaptation and fitness within the dynamic intestinal environment.

**TABLE 1:** Characterized sRNAs in gut commensals

sRNA	Species	Type	Physiological role	Reference
RteR	*B. thetaiotaomicron*	Trans-acting intergenic	Represses conjugative CTnDOT transfer	(74)
DonS	*Bacteroides fragilis*	Cis-antisense	Represses expression of an *N*-glycan polysaccharide utilization locus	(75)
GibS	*B. thetaiotaomicron*	Trans-acting intergenic	Coordinates carbohydrate metabolism in response to GlcNAc	(44)
BatR	*B. thetaiotaomicron*	Trans-acting intergenic	Regulates bile acid susceptibility by remodeling the cell surface	(82)
MasB	*B. thetaiotaomicron*	Trans-acting intergenic	Modulates tetracycline susceptibility	(46)
FopS	*B. thetaiotaomicron*	Family of paralogous sRNAs	Represses translation of glycan transporters to fine-tune polysaccharide utilization; antagonized by RbpB	(47)
SrcF	*Segatella copri*	Trans-acting intergenic	Coordinates responses to complex carbohydrates, including inulin, xylan, and glycogen; regulated by HTCS^Inu^; Δ*srcF* strain exhibits severe competitive fitness defect during intestinal colonization	(45)

## *BACTEROIDES* sRNAS IN INTESTINAL ADAPTATION AND FITNESS

The discoveries and resources described in the previous section have allowed for the characterization of several new *Bacteroides* sRNAs relevant to intestinal colonization. GibS (GlcNAc-induced *Bacteroides* sRNA), a 145-nucleotide intergenic sRNA discovered in the transcriptome map generated by Ryan et al. (44), was the first *Bacteroides* trans-acting sRNA characterized since RteR was identified nearly a decade earlier. GibS expression is induced by *N*-acetylglucosamine (GlcNAc), a building block of host mucus glycans. GibS was found to activate expression of an ⍺-galactosidase and β-glucosidase, while repressing genes involved in glycogen synthesis through direct binding to the translation initiation region of the target mRNAs. Although the GibS fitness contribution has not been evaluated *in vivo*, a Δ*gibS* mutant displayed modified growth on GlcNAc, supporting a role for this sRNA in coordinating metabolic adaptation to nutrient availability. As host-derived glycans become increasingly important carbon sources during periods of dietary fiber limitation (22), regulation of carbohydrate metabolism by GibS may contribute to the metabolic flexibility required for persistence within the intestinal environment.

This paradigm has since directly linked sRNA-mediated PUL regulation to *B. thetaiotaomicron* fitness in the murine gut. Using CLIP-seq to define the *in vivo* RNA interactome of the global RNA-binding protein RbpB, Rüttiger et al. identified a previously unrecognized family of 14 paralogous sRNAs, termed FopS (Family of Paralogous sRNAs), that repress translation of specific SusC-family TonB-dependent glycan transporters by base pairing within the 5′ UTRs of their target mRNAs (47). This repression prevents unnecessary synthesis of carbohydrate uptake systems when their cognate substrates are unavailable. RbpB antagonizes FopS-mediated repression, enabling rapid restoration of glycan PUL translation. Consistent with the importance of this regulatory network *in vivo*, deletion of *rbpB* significantly impaired *B. thetaiotaomicron* fitness during colonization of mice maintained on a low-fiber diet, where the bacterium relies on host-derived mucus glycans rather than dietary polysaccharides for growth.

The functional repertoire of *Bacteroides* sRNAs extends beyond carbohydrate metabolism to encompass additional environmental challenges encountered within the gastrointestinal tract. When integrating their transcriptomic data with genome-wide mutant fitness data sets, Ryan et al. identified the sRNA MasB as a regulator of tetracycline susceptibility, providing one of the first direct links between an individual *Bacteroides* sRNA and antibiotic adaptation (46). Notably, RteR represses transfer of the conjugative transposon CTnDOT, which confers tetracycline resistance and whose conjugative transfer is induced by tetracycline exposure (74). *Bacteroides* sRNAs have therefore been implicated in both antibiotic susceptibility and the dispersion of antibiotic resistance genes (28). More recently, the development of a genome-wide CRISPR interference (CRISPRi) platform for systematic sRNA knockdown has also identified the previously uncharacterized sRNA BatR as a determinant of bile susceptibility in *Bacteroides* through the regulation of genes involved in cell surface structure (82). Although only a small fraction of the hundreds of predicted *Bacteroides* sRNAs have been functionally characterized, those identified thus far converge on a common theme: facilitating adaptation to the diverse challenges encountered within the intestinal environment. Whether responding to shifts in available carbon sources, transitioning from dietary polysaccharides to host-derived mucus glycans, altering susceptibility to antibiotics, or remodeling the cell surface during bile exposure, sRNA-mediated regulation provides *B. thetaiotaomicron* with a rapid and energetically efficient means of optimizing gene expression to maximize fitness in the gut. While the contributions of regulators such as MasB and BatR to intestinal colonization have yet to be evaluated directly *in vivo*, the environmental cues they respond to, including antibiotic exposure (83, 84) and bile acids (85, 86), represent physiologically relevant stresses that commensal bacteria must continually navigate to persist within the gastrointestinal tract. Although *B. thetaiotaomicron* has served as the primary model for understanding sRNA biology in gut commensals, recent studies suggest that sRNAs contribute to commensal adaptation and fitness beyond this model species (45).

## BEYOND *BACTEROIDES*: sRNA REGULATION IN A NON-MODEL MICROBIOTA MEMBER

In addition to the foundational studies in *Bacteroides*, sRNAs have also been identified as essential regulatory factors for intestinal colonization beyond this model genus. Chief among these is the *Segatella* RNA colonization factor (SrcF) in *Segatella copri* (45). Although underrepresented in Westernized populations, *S. copri* is present in the intestinal microbiome of 39% of individuals (87). When present, it is highly abundant, being the most abundant species in 34% of individuals (87, 88). This prevalent and abundant species has been associated with inflammatory disease states (45, 89–91) as well as glucose intolerance and insulin resistance (92). Conversely, *S. copri* has been linked to improved glucose and insulin tolerance in the context of a fiber-rich diet (93, 94). Thus, understanding how *S. copri* regulates its activity in response to host diet and consequent nutrient availability in the gut has direct relevance to human health. Using a dRNA-seq pipeline similar to that employed by Ryan et al. for *B. thetaiotaomicron*, El Mouali et al. identified 63 putative sRNAs in the *S. copri* isolate HDD04 and validated several of them via Northern blot analysis (45). The majority of these sRNAs were well-conserved among *S. copri* strains, including the 272-nucleotide intergenic SrcF. El Mouali et al. found that SrcF is conserved in 97% of the *S. copri* strains analyzed and is highly expressed in the metatranscriptomes of humans carrying *S. copri*, suggesting that the sRNA offers an important fitness contribution in the gut. Indeed, while a Δ*srcF* mutant strain exhibited no gross growth defect in rich medium *in vitro*, in mice co-colonized with OMM12 (95), the wild-type strain outcompeted the deletion strain by more than 1,000-fold by day 14 (45). This remarkable phenotype, resulting from the deletion of a single 272-nucleotide noncoding RNA, illustrates the substantial influence that sRNA-mediated regulation can exert on commensal fitness. Gene expression analysis between the wild-type and Δ*srcF S. copri* strains revealed that SrcF differentially regulates *S. copri* PULs, as well as substrate uptake and metabolism, indicating a potential role for SrcF in regulating carbon metabolism. El Mouali et al. observed that SrcF expression is modulated by complex carbohydrates utilized by *S. copri*, including inulin, xylan, and glycogen. The hybrid two-component system regulator of inulin metabolism, HTCS^Inu^ (96), was found to repress SrcF expression in response to inulin and its metabolic derivatives.

Taken together, the characterization of SrcF provides one of the most comprehensive examples of an sRNA governing gut colonization in a human commensal. Following its identification by dRNA-seq and validation by Northern blot, SrcF was shown to be highly conserved, abundantly expressed in human gut metatranscriptomes, and essential for competitive fitness during intestinal colonization. Mechanistically, SrcF integrates information about carbohydrate availability through HTCS^Inu^-dependent sensing of fructan-derived metabolites, allowing *S. copri* to coordinate expression of genes involved in polysaccharide utilization, nutrient acquisition, and metabolism. Notably, SrcF regulation is influenced not only by substrates directly metabolized by *S. copri* but also by polysaccharide degradation performed by neighboring community members, demonstrating that sRNA-mediated regulation can respond to both dietary inputs and microbiota-derived metabolic activities. Despite these advances, the direct mRNA targets of SrcF and the molecular mechanisms by which it modulates PUL expression and carbon metabolism remain incompletely defined. However, the dramatic colonization defect resulting from the deletion of SrcF underscores the importance of post-transcriptional regulation in shaping microbial fitness and highlights how sRNAs can serve as critical intermediaries linking environmental sensing to successful host colonization.

## FUTURE DIRECTIONS

The foundational work in *Bacteroides* has established a powerful framework for investigating how sRNA regulators contribute to commensal adaptation within the dynamic intestinal environment. Genome-wide sRNA maps, such as those available through Theta-Base (44, 46), provide an invaluable resource for identifying candidate regulatory RNAs, yet their potential has only begun to be realized. The recent integration of Theta-Base with fitness data sets will facilitate the discovery of additional sRNA-mediated regulatory networks that promote commensal resilience (46). Such approaches will be particularly informative when examining bacterial responses to conserved features of the inflamed gut, including iron limitation, elevated oxygen tension, and altered nutrient availability (17, 97, 98). Iron homeostasis represents a compelling example. In many bacterial species, the sRNA RyhB is a master regulator of the iron starvation response (32, 43, 54, 57). Although iron acquisition is essential for *Bacteroides* persistence during intestinal inflammation (99–101), no RyhB homolog has been identified in this genus. In other bacterial genera lacking RyhB homologs, including *Pseudomonas* (102), *Neisseria* (103), and gram-positive *Bacillus* (104), functional analogs of RyhB (105) (iron-responsive sRNAs involved in mediating bacterial iron homeostasis) have been identified, suggesting that evolutionarily distinct sRNAs fulfill analogous regulatory functions. Identifying these regulators will provide important insight into how commensals adapt to inflammatory stress while maintaining intestinal colonization.

Equally important will be extending the experimental and computational strategies developed in *Bacteroides* to the many abundant but genetically underexplored members of the gut microbiota. Computational comparative genomic analysis in *Bacteroides* has indicated that sRNAs are often narrowly conserved, with many species- or even strain-specific (106). While broad conservation is a strong indicator of functionality, limited conservation alone is insufficient to rule out specific sRNA regulators. Although SrcF is conserved only among *S. copri* strains, this sRNA still exerts a strong fitness effect in the murine gut (45). The characterization of SrcF demonstrates that systematic sRNA discovery can uncover regulators that profoundly affect competitive intestinal colonization and metabolic adaptation. Similar efforts in other commensals are likely to reveal additional regulatory paradigms. For example, several putative sRNAs have been identified in *Bifidobacterium* species (107, 108), but these candidates remain largely unvalidated, and their contributions to bacterial fitness *in vivo* are untested. Expanding functional studies across phylogenetically diverse commensals will determine whether the principles established in *Bacteroides* represent conserved features of gut bacterial adaptation or lineage-specific regulatory strategies.

Applying such sRNA functional studies to additional commensal species will require several technical considerations. First, these studies will generally require culturable and genetically tractable isolates. Recent advances in culturomics have substantially expanded the diversity of gut bacteria that can be isolated (109–113), and new genetic tools are increasing the number of previously intractable commensals that can be manipulated experimentally (114–117). Second, careful consideration of RNA extraction and library preparation will be essential, as many commonly used RNA purification protocols can result in the loss or under-representation of low-molecular-weight RNAs (118). Protocols (44) or commercial kits that retain the sRNA fraction should therefore be used when profiling sRNAs. However, as has been previously noted, small size alone is not sufficient to identify a transcript as noncoding (77). Increasing evidence that very small proteins, including those encoded by open reading frames (ORFs) of fewer than 50 amino acids, are missed by conventional genome annotations highlights the need to distinguish regulatory sRNAs from small protein-coding transcripts (119, 120). Moreover, a growing number of dual-function sRNAs have been shown to encode small proteins in addition to their regulatory RNA roles (121–125). sRNA screens could therefore be complemented by ribosome profiling (126) to distinguish actively translated small ORFs from strictly noncoding transcripts, thereby improving genome annotation (77). Finally, because sRNA expression can be highly condition- and environment-dependent (44, 127), profiling across physiologically relevant growth conditions will be important for capturing fitness-relevant sRNAs. Collectively, these considerations will be critical for identifying and functionally validating fitness-relevant sRNAs across non-model gut commensals.

Beyond their roles in promoting commensal fitness, sRNAs may also provide critical insight into the mechanisms by which normally beneficial bacteria transition to pathobionts under specific environmental conditions. Dietary fiber deprivation drives *B. thetaiotaomicron* to shift from consuming dietary polysaccharides to degrading host mucus glycans, thereby eroding the intestinal mucus barrier and increasing susceptibility to enteric infection and graft-versus-host disease (22, 23). As several sRNAs have already been implicated in regulating PULs in *Bacteroides* (44, 47, 75, 81), defining these regulatory networks may reveal how post-transcriptional regulation contributes to this consequential shift in metabolic behavior. Likewise, the effects of *S. copri* on host physiology appear to depend on dietary context (94, 96). The diet-dependent *Segatella* sRNA, SrcF, is downregulated by fructooligosaccharides. Moreover, these sugars modulate colonization (128), competition (129), and cooperation (130) among *Bacteroides*. Thus, SrcF could exert a powerful influence on *S. copri’s* interbacterial interactions and overall microbiota stability. Similar questions extend to opportunistic organisms such as *Fusobacterium nucleatum*. Normally an oral commensal, intestinal *F. nucleatum* colonization has been associated with inflammatory disease and the development of colorectal cancer (131–134). Recently, *F. nucleatum* has been shown to upregulate the sRNAs FoxI and FoxJ, which mediate its envelope stress response, upon exposure to oxygen and bile, two environmental stressors encountered during intestinal colonization (135). Additionally, the RNA-binding proteins KhpA and KhpB, which are associated with FoxI-mediated regulation, are required for ethanolamine metabolism in *F. nucleatum* (136). Ethanolamine is an abundant intestinal metabolite that is critical for fusobacterial virulence (137), suggesting that sRNA regulation may facilitate the metabolic and stress adaptations required for adaptation to virulence-associated, extra-oral colonization sites. Together, these observations suggest that sRNA regulatory networks may influence not only commensal resilience but also the environmental transitions that underlie pathobiont behavior. Defining these mechanisms will deepen our understanding of host-microbe interactions and may ultimately reveal new targets for therapeutic modulation of the gut microbiota.

## CONCLUDING REMARKS

Over the past decade, small RNA regulators have emerged from being an underappreciated component of bacterial gene regulation to recognized determinants of microbial adaptation and fitness. Although our understanding of bacterial sRNAs has largely been shaped by studies of model organisms and pathogens, recent work has demonstrated that these post-transcriptional regulators also play a central role in the biology of gut commensals. Much of this progress has been driven by studies of *B. thetaiotaomicron*, in which systematic transcriptome mapping, genetic manipulation, and mechanistic characterization have established a framework for identifying and characterizing sRNAs that regulate nutrient acquisition, stress responses, and host colonization. These studies have illustrated how sRNAs integrate environmental signals to drive rapid changes in gene expression, enabling *Bacteroides* to respond efficiently to the highly dynamic conditions of the intestinal ecosystem.

Importantly, emerging work beyond *Bacteroides* suggests that these regulatory principles are not restricted to a single genus. The discovery of SrcF in *Segatella copri* demonstrates that individual sRNAs can exert profound effects on competitive intestinal colonization by coordinating metabolic responses to nutrient availability. Given the enormous phylogenetic diversity of the gut microbiota, however, these examples likely represent only a small fraction of the sRNA-mediated regulatory networks that shape microbial fitness. The vast majority of commensal sRNAs remain unexplored, leaving fundamental questions unanswered regarding the prevalence, conservation, and evolution of these regulatory systems across the gut ecosystem.

Future studies should therefore focus on extending the experimental and computational approaches pioneered in *Bacteroides* to a broader diversity of gut microorganisms. Advances in dRNA-seq (76, 77), comparative genomics (106, 138, 139), and RNA interactome mapping (140–143) now make it feasible to identify sRNAs and define their regulatory targets in previously inaccessible commensal species. Applying these approaches across diverse taxa will reveal whether common regulatory strategies govern adaptation to the intestinal environment or whether distinct microbial lineages have evolved unique RNA-based mechanisms to occupy specialized ecological niches. Integrating sRNA biology with metatranscriptomics (144), metabolomics (145), and microbiome community models (146, 147) will further provide insight into how these regulators coordinate microbial interactions and responses to changing host environments. Such knowledge may ultimately identify new opportunities to manipulate commensal gene regulation for therapeutic modulation of the gut microbiota in health and disease.
